# Assessing the Adequacy of Traditional Vertebral Landmarks as Upper Border of Whole Pelvic Radiotherapy Field for Stage IB2-IIB Cervical Cancer

**DOI:** 10.3390/cancers16152743

**Published:** 2024-08-01

**Authors:** Ji Hwan Jo, Jeong Won Lee, Ki Ho Seol

**Affiliations:** 1 Department of Radiation Oncology, Daegu Catholic University Medical Center, Daegu 42472, Republic of Korea; fiery2004@naver.com (J.H.J.); gardenlee@cu.ac.kr (J.W.L.); 2 Department of Radiation Oncology, Daegu Catholic University School of Medicine, Daegu 42472, Republic of Korea

**Keywords:** uterine cervical neoplasms, radiotherapy, lymph nodes

## Abstract

**Simple Summary:**

Traditionally, the upper border of whole pelvic radiotherapy fields for cervical cancer was set using vertebral landmarks, potentially missing a part of the common iliac lymph node (CIN). This retrospective study investigated the impact of insufficient radiation coverage of CIN in stage IB2-IIB cervical cancer treatment. We compared outcomes between patients with full CIN coverage and those with partial coverage. Our results showed that patients with full CIN coverage had significantly better outcomes, including lower recurrence rates and higher survival rates. These findings suggest that using vascular anatomy (aortic bifurcation) as a guide for setting the upper border of radiation fields may be more effective than relying on vertebral landmarks. Our study highlights the importance of ensuring comprehensive coverage of the CIN area in cervical cancer treatment, which may influence future radiotherapy practices by encouraging the use of vascular landmarks for field border definition.

**Abstract:**

This study investigates the impact of insufficient common iliac lymph node (CIN) irradiation on treatment outcomes in patients with stage IB2-IIB cervical cancer receiving concurrent chemoradiotherapy (CCRT). We retrospectively analyzed 68 patients with Federation of Gynecology and Obstetrics stage IB2-IIB, treated with weekly cisplatin-based CCRT from 2008 to 2018. Patients received external-beam whole pelvic radiotherapy (WPRT) and concurrent cisplatin chemotherapy, followed by high-dose-rate brachytherapy. The WPRT upper border was at L4-5 in 61 patients and L3-4 in 7 patients. Thirty-seven patients had the CIN area fully included (full-CIN group), while 31 had partial inclusion (partial-CIN group). Recurrence rates and survival outcomes were analyzed over a median follow-up of 111 months. Patient characteristics and the irradiated dose were comparable. Treatment failure occurred in three patients (8.1%) in the full-CIN group and in six patients (19.4%) in the partial-CIN group, with CIN and para-aortic lymph node recurrence in two and one patients, respectively. The 5-year cumulative recurrence rate was 0% for the full-CIN group and 11.4% for the partial-CIN group (*p* = 0.04). Cause-specific survival was 100% vs. 87.1% (*p* = 0.025), and the overall survival was 94.3% vs. 87.1% (*p* = 0.44). Fully including the CIN area in WPRT is crucial for stage IB2-IIB cervical cancer. Vascular anatomical margins should be considered over vertebral landmarks.

## 1. Introduction

Cervical cancer is the fourth most common cancer in women globally and the fourth leading cause of cancer-related deaths in women, with an estimated 661,021 new cases and 348,189 deaths worldwide in 2022 [1]. In patients with locally advanced cervical cancer, concurrent chemoradiotherapy (CCRT) has shown improved overall survival compared with radiotherapy alone and is currently used as the standard treatment [2,3,4,5]. However, despite the implementation of CCRT, locoregional recurrence still frequently occurs. Eifel et al. reported that after CCRT, the 8-year locoregional recurrence rate was 16%, and the 8-year para-aortic recurrence rate was 9% [6]. These locoregional recurrence rates underscore the need for the adequate coverage of regional lymph node areas with radiotherapy.

The spread of cervical cancer may progress to the obturator lymph nodes (LNs), which are considered a medial group of the external iliac chain, other external iliac LNs, and hypogastric LNs. From these, there may be tumor metastases to the common iliac or para-aortic LNs [7,8]. The risk of nodal metastases increases per the International Federation of Gynecology and Obstetrics (FIGO) stage, with incidences in the pelvic region ranging from 2% (stage IA2) to 14–36% (IB), 38–51% (IIA), and 47% (IIB); and in the para-aortic region ranging from 2 to 5% (stage IB), 10–20% (IIA), and 9% (IIB) [9]. Based on this, the current guidelines for stage I-II cervical cancer endorse the nodal region-based clinical target volume recommendations. Whole pelvic radiotherapy (WPRT) should include the obturator, internal, external, presacral, and common iliac LNs [10,11,12].

A recent survey indicated that in South Korea, for the radiotherapy of stage I-IIB cervical cancer, the ‘upper border of the radiotherapy field’ is commonly set at the L4-5 level or the sacral promontory [13]. Notably, only 17.2% responded that they based it on the common iliac artery rather than on the vertebral bone landmarks. Furthermore, rather than identifying the aortic bifurcation as the upper border of the WPRT field, the upper border was determined by setting the vertebrae as landmarks based on traditional two-dimensional radiotherapy. Aortic bifurcation is apparent at the L4–5 interspace in 70–93% of patients based on previous radiologic anatomy studies; however, determining the upper border using the vertebrae as a landmark according to the traditional practice of two-dimensional radiotherapy may result in the insufficient irradiation of the common iliac LN (CIN) area [14,15,16,17]. This causes a gray zone between the upper border of the radiation field and the aortic bifurcation in some patients.

There is insufficient research on the impact of inadequate irradiation on the CIN area, which can occur when the upper border of the WPRT is determined using the L4-5 junction or sacral promontory as bony landmarks in patients with cervical cancer IB2-IIB undergoing CCRT. Therefore, we aimed to investigate the effect of insufficient CIN irradiation on the treatment outcomes of patients with stage IB2-IIB cervical cancer receiving CCRT. This could help determine whether traditional vertebral landmarks or vascular anatomy should be used to set radiation fields, especially in clinical practice where intensity-modulated radiotherapy (IMRT) is increasingly being utilized.

## 2. Materials and Methods

### 2.1. Patients

This retrospective study was approved by the Institutional Review Board of the Daegu Catholic University Medical Center (IRB No. CR-23-167-L). The inclusion criteria were as follows: (1) newly diagnosed histologically proven squamous cell carcinoma, adenocarcinoma, or adenosquamous carcinoma of the uterine cervix at our institution between 2008 and 2018; (2) clinical and radiologic FIGO stage IB2-IIB with no other evidence of pelvic LN involvement or distant metastasis; (3) treatment using a combination of computed tomography (CT)-based three-dimensional conformal external-beam radiotherapy (3D-CRT) and, concurrent cisplatin-based chemotherapy, followed by high-dose-rate (HDR) brachytherapy; and (4) Eastern Cooperative Oncology Group performance status 0–2. The exclusion criteria were as follows: (1) incomplete treatment, (2) surgical intervention or other treatment before CCRT, (3) a history of cancer in another organ, (4) incomplete clinical information, and (5) positive pelvic or para-aortic LN (PAN) metastasis diagnosed through imaging or biopsy before CCRT.

Finally, we retrospectively analyzed 68 patients with FIGO stage IB2-IIB treated with weekly cisplatin-based CCRT at a single institution between 2008 and 2018. CT images for radiotherapy planning were analyzed, and the upper border of the WPRT and the location of the aortic bifurcation for all patients were confirmed. All patients were divided into two groups: the full-CIN group (the CIN area sufficiently included in the irradiation field) and the partial-CIN group (the irradiation field did not sufficiently cover the CIN area) (Table 1).

### 2.2. Treatment

Notably, all patients received a combination of external-beam WPRT using CT-based 3D-CRT and concurrent cisplatin-based chemotherapy (weekly cisplatin regimen), followed by HDR brachytherapy. External-beam radiation therapy (EBRT) was delivered using a linear accelerator (Varian Clinac 21EX (Varian Medical System, Palo Alto, CA, USA)) with four fields (anteroposterior, posteroanterior, and two lateral fields) of 6–10 MV photons or with anteroposterior–posteroanterior opposed beams of 10 MV photons. As required, the field-in-field technique, which involves manually adjusting the multileaf collimator field to generate subfields, was used to reduce the hotspot size or improve the homogeneity index. All patients received a median EBRT dose of 45 Gy (range: 39.6–54 Gy) at 1.8 Gy per fraction to the whole pelvic area. For patients with parametrium involvement, a boost irradiation of a median 9 Gy (range: 5.4–10 Gy) at 1.8 Gy or 2 Gy per fraction was administered to the parametrium area based on the radiation oncologist’s assessment. The decision to reach the upper border of the WPRT was made at the radiation oncologist’s discretion. The L4-5 interspace is the most frequently used upper border. In some cases, the L3-4 interspace was determined to be the upper border to sufficiently include the CIN area. After adequate tumor regression, HDR brachytherapy was performed twice weekly using the iridium-192 remote after-loading technique. HDR brachytherapy plans for cervical cancer were generated using two-dimensional (2D) orthogonal x-ray films (Point A-based 2D conventional brachytherapy). Patients were treated with HDR brachytherapy using a Fletcher–Williamson applicator (Nucletron B.V., Veenendaal, The Netherlands) or Henschke applicator (Nucletron B.V., Veenendaal, The Netherlands). A dose of 5 Gy was prescribed to point A in all the patients. The doses to the bladder and rectum points were maintained below 75% of the dose to point A. The standard prescribed dose for each brachytherapy session in our institution was 5.0 Gy to the A-point in six fractions, twice weekly. The median prescribed A-point dose was 30 Gy (range: 25–35 Gy). The combined total dose from EBRT and HDR brachytherapy was calculated using a linear quadratic model to determine the radiobiological equivalent dose in 2 Gy fractions (EQD2) (α/β = 10) [18]. The mean total prescribed A-point EQD2 was 84.98 Gy. Cisplatin-based chemotherapy was administered concurrently with EBRT in all patients. Chemotherapy was initiated at the start of WPRT. During radiotherapy, cisplatin chemotherapy (40 mg/m^2^ weekly for 6 weeks) was administered to all patients. The chemotherapeutic dose was reduced in patients with severe hematological or non-hematologic toxicity.

### 2.3. Comparison Group, Endpoints, and Statistical Methods

The CIN and PAN recurrence rates and survival outcomes were analyzed in both groups. The primary endpoints for treatment comparison were the 5-year CIN and PAN recurrence, disease-free survival (DFS), cause-specific survival (CSS), and overall survival (OS) rates. DFS was defined as survival without locoregional, CIN, PAN, or distant recurrences. In-field pelvic recurrence was defined as the recurrence or progression of the primary tumor or pelvic (regional) LN failure in the irradiated pelvic region. Out-of-field CIN and PAN recurrence was defined as a recurrence of CIN or PAN outside the irradiation field. We considered a persistent cervical disease that did not regress for 3 months after completing radiotherapy as in-field pelvic recurrence. Distant metastasis was defined as a recurrence outside the infrarenal para-aortic field. For disease events, we considered the first sites of recurrence as disease sites. We calculated all occurrences from the date of diagnosis to the date of relapse or last follow-up. Deaths due to other causes were censored at the final follow-up.

The chi-square test, Fisher’s exact test, and an independent *t*-test were used to analyze differences between groups. Survival analysis was based on the Kaplan–Meier method, and the results were compared using the log-rank test and Cox regression analysis. All analyses were performed using the SPSS ver. 19.0 (IBM, Armonk, NY, USA), and the statistical significance was set at *p*-value < 0.05.

## 3. Results

### 3.1. Patient Characteristics

Overall, 68 patients were included in the analysis. The upper border of the WPRT (vertebral landmark) was the L4-5 interspace level in 61 patients and the L3-4 interspace level in 7 patients.

**Figure 1:**
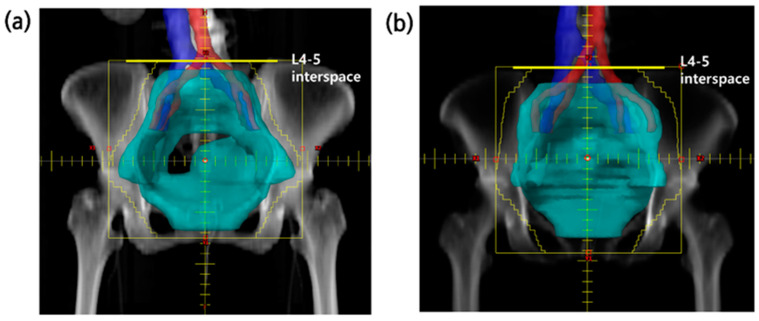
Patients in the full and partial-CIN groups: example of beam’s eye view. (**a**) The aortic bifurcation is located at the upper border (yellow line) of WPRT. (**b**) The aortic bifurcation is located outside the upper border (yellow line) of WPRT.

Notably, 37 of the 68 patients had a CIN area that was sufficiently included in the irradiation field (full-CIN group). However, in 31 patients, the CIN was not sufficiently covered by the irradiation field (partial-CIN group). There were no cases where the irradiation field completely excluded the CIN area. Figure 1 shows the beam’s eye-view images of each group. Except for the upper border of the WPRT, no significant differences were observed between the groups regarding patient characteristics or treatment, including irradiation dose (Table 1).

### 3.2. Clinical and Survival Outcomes

The median follow-up period was 111 (interquartile range: 57–125.5), 114, and 106 months for all patients, those in the full-CIN group, and for those in partial-CIN groups, respectively. Notably, all patients achieved complete remission at the 3-month examination after CCRT.

The incidence of out-of-field CIN and PAN recurrence was higher in the partial-CIN group. Treatment failure was observed in three patients (8.1%) in the full-CIN group. One case of in-field vaginal recurrence and two cases of distant lung metastases occurred; however, CIN–PAN recurrence was not observed. In the partial-CIN group, treatment failure occurred in six patients (19.4%), of which two had CIN recurrence (out-of-field) and one had PAN recurrence. Among the patients in the partial-CIN group, one had local in-field recurrence, and one had distant metastasis. Local recurrence and distant metastases were simultaneously observed in the remaining patients. Table 2 lists the detailed failure patterns.

**Figure 2:**
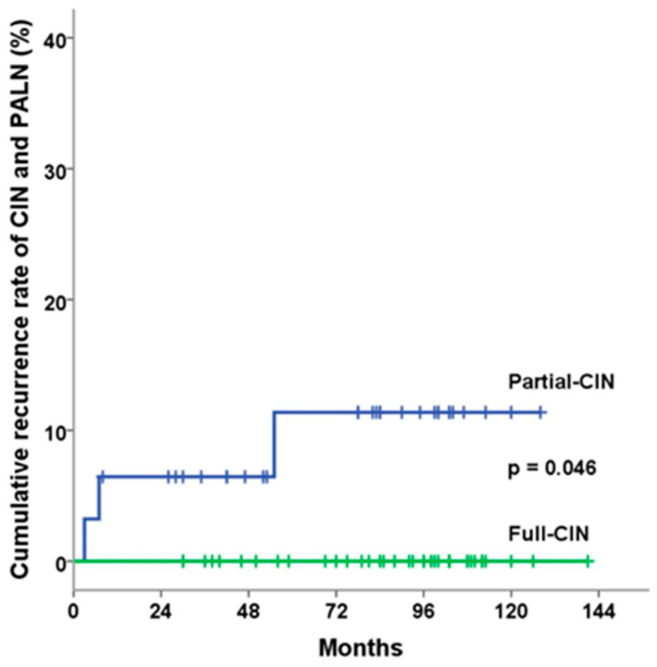
Cumulative rate of CIN and PAN recurrence as classified based on the radiotherapy field. The 5-year cumulative recurrence rate of CIN and PAN in the full and partial-CIN groups was 0% and 11.4% (*p* = 0.04), respectively.

**Figure 3:**
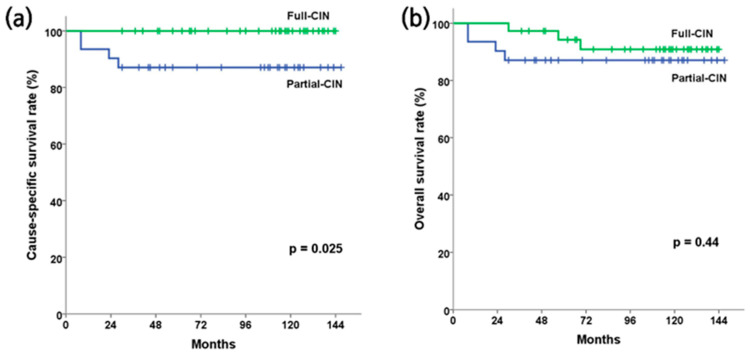
Survival outcome estimation using Kaplan–Meier analysis. (**a**) Cause-specific survival rate, (**b**) overall survival rate. The 5-year cause-specific and overall survival rates in the full and partial-CIN groups were 100% vs. 87.1% (*p* = 0.025) and 94.3% vs. 87.1% (*p* = 0.44), respectively.

Regarding the cumulative recurrence rate of CIN and PAN as well as cause-specific survival, the full-CIN group demonstrated statistically significant better outcomes. Figure 2 and Figure 3 illustrate the Kaplan–Meier curves according to CIN coverage. The 5-year cumulative recurrence rates of CIN and PAN in the full and partial-CIN groups were 0% and 11.4% (*p* = 0.04), respectively (Figure 2). The 5-year cause-specific survival and overall survival rates for the full and partial-CIN groups were 100% vs. 87.1% (*p* = 0.025) and 94.3% vs. 87.1% (*p* = 0.44), respectively (Figure 3).

The most common acute toxicities were gastrointestinal complications. Although overall grade 1–2 acute side effects were often observed during CCRT, they were generally self-limiting or resolved with medical management. Due to acute complications, including neutropenia and/or concerns about potential toxicity, two patients (5.4%) in the full-CIN group and two patients (6.4%) in the partial-CIN group were treated with a reduced dose of the chemotherapeutic agent during CCRT. No other grade 3 or higher acute complications were observed. Grade 3 or higher late complications were reported in a total of 11 patients: 6 patients (16.2%) in the full-CIN group and 5 patients (16.1%) in the partial-CIN group. In the full-CIN group, there were five patients with grade 3 proctitis and one patient with grade 3 colitis. In the partial-CIN group, three patients had grade 3 proctitis, one had grade 3 bowel obstruction, and one had grade 4 bowel obstruction. Both groups showed no significant difference in treatment toxicity.

## 4. Discussion

Pelvic lymph node metastasis is common in patients with cervical cancer [7,8,19,20], and this is an essential prognostic factor for treatment outcomes. One study examined pelvic lymph node involvement in 225 patients with cervical cancer who underwent radical hysterectomy, and 14.2% of patients with stage IB and IIA disease had positive pelvic lymph nodes. This rate was 19.8% for patients with stage IIB cancer and 28% for those with stage IIIB cancer. The lymph node groups with the most frequent metastases were the parametrial, obturator, external iliac, and common iliac chains. Para-aortic lymph node metastases were present in 3.3% of stage IB/IIA cases with tumors ≤4 cm, whereas 13.1% of patients with stage IIB/III had para-aortic involvement [19]. Therefore, these lymph node areas should be adequately covered when performing radiotherapy.

This retrospective study investigated the impact of the insufficient coverage of the CIN area during WPRT in patients with stage IB2-IIB cervical cancer treated with CCRT. Our critical finding was that patients whose WPRT plans fully encompassed the CIN region (full-CIN group) experienced significantly better outcomes than those with incomplete CIN coverage (partial-CIN group). At 5 years, the full-CIN group had 0% CIN/para-aortic recurrence compared with the 11.4% in the partial-CIN group (*p* = 0.04). Moreover, the cause-specific survival rate was 100% for the full-CIN group but only 87.1% for those who received suboptimal CIN irradiation (*p* = 0.025). These results likely stem from the fact that when the upper WPRT border was set using vertebral landmarks like L4-L5 per conventional practice, a portion of the CIN area could be inadvertently excluded from the treatment fields in some patients. Therefore, any lymph node metastasis present in this underdosed region would be treated inadequately, increasing the risk of regional recurrence.

Our findings align with those of previous studies, emphasizing the importance of tailoring radiation fields to encompass lymph node regions at risk of harboring metastases based on each patient’s disease stage and extent. Previous studies have reported that the radiation field in conventional four-field RT based on bony landmarks may be inappropriate [14,15,16,17,21,22,23,24,25,26,27,28]. One study suggested that vessel-contouring-based individualized field design can provide adequate nodal coverage [29].

Notably, some studies have reported that when the upper border of the WPRT is determined using vertebral landmarks like L4-L5 per conventional practice, a portion of the common iliac node area may be inadvertently underdosed in some patients, increasing the risk of regional recurrence [30,31,32]. Zhang et al. assessed pelvic lymph node coverage using conventional radiation fields based on bony landmarks in patients with cervical cancer. They used 100 patients with cervical cancer who underwent CT simulation to contour the pelvic vessels as surrogates for lymph node location. The distances were measured between conventional pelvic field borders and key vessel locations (bifurcation of the aorta and distal external iliac arteries) to assess lymph node coverage adequacy. Notably, most inadequacies were at the superior border, not covering the common iliac nodes, and at the lateral borders, not covering the distal external iliac nodes. Therefore, they concluded that the pelvic lymph node coverage of conventional radiation fields based on bony landmarks may be inadequate in patients with cervical cancer [30]. Beadle et al. analyzed the regional recurrence patterns in 198 patients with cervical cancer treated with definitive radiotherapy. Notably, most patients received radiotherapy alone, and the superior borders of the radiation fields varied widely, ranging from as low as S2/3 to as high as T12-L1 or higher, depending on the extent of lymph node involvement.

Furthermore, 119 (66%) of 180 patients with a documented location of recurrence had a component of marginal failure occurring outside the radiation field, most commonly above the radiation field (71 patients), and 105 (58%) had a component of in-field failure within the radiation field. They found that of the 68 patients who had recurrences above the radiation field and had regional imaging available at diagnosis, most (63%) initially showed no evidence of lymph node involvement. These recurrence patterns suggest potential deficiencies in properly defining the target volume [31]. Rai et al. reported that when the upper border of radiotherapy was determined based on the vertebral landmarks between L3 and S1, only 29% of 116 patients with cervical cancer received adequate irradiation in the CIN area. Furthermore, it was reported that more than half of the recurrences outside the radiation field occurred between the aortic bifurcation and the L4-5 interspace. They indicated that the insufficient irradiation of the CIN area may have an adverse effect on local tumor control [32].

The higher regional recurrence rates and associated decrease in cancer-specific survival observed in our partial-CIN cohort underscore the need to ensure comprehensive CIN coverage during WPRT for locally advanced cervical cancer. Rather than relying on traditional vertebral body landmarks, which can be misleading, our study suggests that vascular anatomy (aortic bifurcation level) can provide a more reliable surrogate for determining the appropriate WPRT superior border, especially with modern conformal radiotherapy techniques such as IMRT, that enable accurate targeting. Therefore, adopting this approach could allow for maximal therapeutic effects by adequately encompassing all areas at risk of harboring lymph node metastases based on cross-sectional imaging and established patterns of cervical cancer spread.

In our study, patients with full CIN coverage demonstrated excellent outcomes, with a 5-year cause-specific survival and overall survival rates of 100% and 94.3%, respectively. These results are comparable to those reported in the EMBRACE-I study [33], although a direct comparison is challenging due to the use of different staging systems (the EMBRACE-I used FIGO 2009). The EMBRACE-I reported a 5-year overall survival of 79% to 88% for FIGO stage IB-IIB patients without nodal involvement at diagnosis. Regarding nodal control, our study showed no out-of-field CIN and PAN recurrence in the full-CIN group, which aligns with the favorable nodal control rates reported in EMBRACE-I. The latter reported a 5-year nodal control rate of 78% to 92% for FIGO IB-IIB patients without nodal involvement. However, it is important to note that in the partial-CIN group, there were three cases of out-of-field CIN and PAN recurrence. This finding, coupled with the fact that PAN recurrence was the major nodal failure pattern in the EMBRACE-I, underscores the potential negative impact of inadequate CIN coverage and supports our conclusion that ensuring full coverage of the CIN area is crucial for optimal outcomes. Our findings on the importance of adequate CIN coverage align with one of the main goals of the ongoing EMBRACE-II study (NCT03617133), which aims to reduce nodal recurrence in the upper common iliac and para-aortic regions. The EMBRACE-II study hypothesizes that enlarging the CTV may reduce nodal recurrence, potentially improving overall survival. This hypothesis awaits validation through the completion and follow-up of the EMBRACE-II study.

This study has several limitations. Firstly, the retrospective nature of the study inherently introduces potential biases. Secondly, due to the relatively small sample size of this study, the statistical power was insufficient. This also limits the generalizability of the results to broader patient populations. Thirdly, the use of older imaging techniques may have led to an underestimation of the lymph node involvement status in some patients. Modern multimodality staging might have provided more accurate lymph node staging, potentially altering patient grouping and subsequent outcomes. Fourthly, since the patients were treated at a single institution, this limits the external validity of the study. Practices may vary between institutions, so the results may not be directly applicable to settings with different treatment protocols or patient populations. Lastly, the long follow-up period, while a strength of the study, may obscure the impact of evolving treatment practices over time on outcomes.

However, the groups were well-balanced, and because the patients were treated at a single institution, the treatment protocol was relatively uniform. Our findings provide compelling evidence that ensuring full CIN coverage during WPRT is critical for optimal locoregional control in patients with stage IB2-IIB cervical cancer. However, despite the study’s limitations, the consistent treatment approach and balanced groups support the importance of adequate target volume coverage to improve outcomes in this patient population.

## 5. Conclusions

Our data indicate that positioning the superior WPRT border at or above the level of the aortic bifurcation should be considered for patients with stage IB2-IIB cervical cancer to ensure the adequate treatment of the CIN region, rather than using vertebral landmarks that may underdose this area. Further prospective studies are warranted to validate these findings and comprehensively investigate field designs that maximize the therapeutic ratio by covering disease-associated lymph node areas at risk while avoiding excessive normal tissue exposure. With IMRT and other advanced techniques, tailoring radiation fields precisely to regions of potential involvement could improve outcomes for locally advanced cervical cancer compared with historical field conventions.

## Figures and Tables

**Table 1:** Characteristics of patient.

Variables	Full-CIN Group	Partial-CIN Group	*p* Value
	N = 37	n = 31	
Upper border of RT (vertebral landmark)			0.011
L3-4 interspace	7	0	
L4-5 interspace	30	31	
Age (years, mean ± SD)	54.8 ± 12.5	53.2 ± 11.8	0.589
Comorbidities			
Hypertension	10	3	0.070
Diabetes mellitus	2	0	0.189
Histopathology			0.742
SCC	30	27	
Adenocarcinoma or ASC	7	4	
FIGO stage			0.959
IB2, IB3	13	12	
IIA	7	4	
IIB	17	15	
Primary tumor size (mm, mean ± SD)	3.63 ± 1.07	3.60 ± 1.03	0.918
Primary tumor size, cm			0.353
<4	21	21	
≥4	16	10	
SUVmax of PET in primary lesion (mean ± SD)	9.78 ± 3.79	9.81 ± 3.74	0.971
Pretreatment hematologic parameter			
Hemoglobin (g/dL, mean ± SD)	12.55 ± 1.50	11.90 ± 1.87	0.116
SCC Ag. Level (ng/mL, mean ± SD)	5.19 ± 4.86	7.27 ± 7.43	0.188

CIN, common iliac lymph node; SD, standard deviation; RT, radiotherapy; SCC, squamous cell carcinoma; ASC, adenosquamous carcinoma; FIGO, The International Federation of Gynecology and Obstetrics; SUV, standardized uptake values; PET, positron emission tomography; SCC Ag., squamous cell carcinoma-related antigen.

**Table 2:** Recurrence pattern according to CIN coverage.

Recurrence Pattern	Full-CIN Group (N = 37)	Partial-CIN Group (N = 31)	*p* Value
In-field pelvic recurrence	1	2	0.588
Out-of-field CIN and PAN recurrence	0	3	0.090
Distant recurrence	2	2	1.000

CIN, common iliac lymph node. One patient in the partial-CIN group had both local recurrence and distant metastasis.

## Data Availability

The data that support the findings of this study are available on request from the corresponding author. The data are not publicly available due to privacy or ethical restrictions.
